# Whole Genome Sequencing Improves the Identification of Pathogenic and Novel Variation in Nonsyndromic Hearing Loss

**DOI:** 10.1155/humu/6371082

**Published:** 2025-12-01

**Authors:** Stefan Rentas, Ramakrishnan Rajagopalan, Tolga Ayazseven, Mahdi Sarmady, Sarah E. Raible, Ian D. Krantz, Ahmad N. Abou Tayoun

**Affiliations:** ^1^Department of Pathology, Duke University School of Medicine, Durham, North Carolina, USA; ^2^Department of Pathology and Laboratory Medicine, Children's Hospital of Philadelphia, Philadelphia, Pennsylvania, USA; ^3^Division of Genetics, Children's Hospital of Philadelphia, Philadelphia, Pennsylvania, USA; ^4^Division of Medical Genetics, Cohen Children's Medical Center, Northwell Health, New York, New York, USA; ^5^Department of Pediatrics, Zucker School of Medicine, Hofstra University, New York, New York, USA; ^6^Dubai Health Genomic Medicine Center, Al Jalila Children's Specialty Hospital, Dubai, UAE; ^7^Center for Genomic Discovery, Mohammed Bin Rashid University of Medicine and Health Sciences, Dubai Health, Dubai, UAE

## Abstract

**Background:**

Genetic testing is essential to the diagnosis of nonsyndromic bilateral sensorineural hearing loss (BSNHL), where pathogenic variants in *GJB2* are the most common cause. Current testing strategies often fail to provide a comprehensive diagnosis and typically require the use of multiple testing methodologies. This study evaluated the diagnostic utility of genome sequencing (GS) in a cohort with heterozygosity for *GJB2* pathogenic variants and BSNHL.

**Methods:**

A retrospective cohort of 23 individuals with BSNHL and a heterozygous pathogenic variant in *GJB2* underwent targeted *GJB2* resequencing and variant reinterpretation. Those without biallelic *GJB2* variants upon single gene reanalysis proceeded to exome sequencing (ES) using a large virtual panel of hearing loss–associated genes. Subjects with no definitive diagnosis from ES subsequently underwent GS. Variants were interpreted using hearing loss–specific ACMG guidelines and published literature.

**Results:**

Three individuals were diagnosed with biallelic pathogenic variants upon *GJB2* single gene reanalysis. ES identified a definitive or likely diagnosis in five different hearing loss–related genes in 5/20 (25%) individuals, while two additional cases remained inconclusive due to novel or ambiguous variants in two other hearing loss–associated genes. GS of the remaining 15 cases yielded diagnoses in three individuals, including the identification of deletions in *LOXHD1* and *STRC*, and a recently characterized 125 kb deletion overlapping *CRYL1*, which refines a critical upstream regulatory region associated with *GJB2*-related hearing loss. Overall, 11/23 (48%) individuals received a diagnosis with our stepwise testing approach, with GS providing sequencing coverage of all findings.

**Conclusion:**

GS improves diagnostic yield in patients with BSNHL, capturing both SNVs and CNVs missed by ES and targeted testing, and supports its adoption as a comprehensive first-tier diagnostic test for nonsyndromic hearing loss.

## 1. Introduction

Approximately 50% of congenital and childhood hearing loss has a genetic origin with the majority of individuals presenting with autosomal recessive nonsyndromic hearing loss [1]. Historically, molecular genetic testing for hearing loss has prioritized sequencing of the *GJB2* (connexin-26) gene, which is the most common genetic cause of congenital, nonsyndromic, mild to profound bilateral sensorineural hearing loss (OMIM #220290) [1]. Pathogenic variants contributing to *GJB2*-related hearing loss can include deleterious missense and truncating variants [2] as well as large deletions upstream of *GJB2*'s coding region that abolish essential regulatory elements necessary for *GJB2* expression [3–6].

Aside from *GJB2*, the remaining landscape of genetic causes of hearing loss is heterogeneous, with curation efforts finding at least 100 definitively or strongly associated genes and around 60% of which display autosomal recessive (ARHL) inheritance [7, 8]. Additionally, copy number variants (CNVs) play a significant role and have been identified in 29 different nonsyndromic hearing loss genes and are involved in approximately 20% of cases [9, 10]. The most frequently encountered CNVs include upstream deletion of *GJB2* and deletions involving *STRC* (stereocilin) and *OTOA* (otoancorin); these latter two genes typically require additional specialized molecular assays to detect copy number alterations since both have pseudogenes with high homology [9]. Thus, laboratories performing genetic testing for hearing loss routinely combine a variety of methodologies, such as single gene sequencing, NGS gene panel, multiplex ligation probe amplification (MLPA), droplet digital PCR (ddPCR), and microarray [11]. Despite the various techniques used to capture pathogenic variants in hearing loss–related genes, at least 30%–50% of nonsyndromic hearing loss cases remain without a genetic diagnosis [12–14]. This can be attributed to a nongenetic origin, uncharacterized disease genes, or a missed pathogenic alteration in a known disease gene.

Genome sequencing (GS) is an appropriate substitute for complex hearing loss testing algorithms as it allows for accurate detection of single nucleotide variants (SNV), small insertion–deletions (indel), coding and noncoding CNVs, and structural variants (SV) and can evaluate variants in candidate genes not previously associated with hereditary hearing loss. Exome sequencing (ES) broadly performs similarly to GS in clinical settings but is unable to detect variants in noncoding regions and copy neutral SVs and can only detect exonic gains and losses from larger CNVs. This distinction is important as ES likely limits the ability to observe rare but clinically important CNVs and SVs in patient genomes. Here, we explore the utility of GS and ES on a historical cohort of hearing loss patients (2003–2011) that received only *GJB2* sequencing and were carriers of a single, apparently pathogenic variant. This cohort was originally chosen to search for novel CNVs within the upstream coding region of *GJB2* and to potentially find the “missing” transvariant in *GJB2* carriers. Using this cohort, we further evaluated alternative causes of hearing loss in other genes, performed candidate gene searches, and compared the diagnostic yield of GS to ES. Overall, our data supports GS as a first-tier test for nonsyndromic hearing loss as it led to additional diagnoses missed by ES, including the identification of a 125 kb deletion upstream of *GJB2*, which further refines the region critical for *GJB2* expression and function.

## 2. Materials and Methods

### 2.1. Human Subjects

The retrospective study cohort included 23 individuals (11 females and 12 males) with bilateral sensorineural hearing loss and an apparently heterozygous pathogenic variant detected in the *GJB2* gene by Sanger sequencing from 2003 to 2011 (Table S1). All subjects were enrolled in an IRB-approved research protocol at Children's Hospital of Philadelphia (IRB 00-002059). All subjects were deidentified.

### 2.2. Large Language Models (LLMs)

Highly edited ChatGPT5 outputs were used for drafting the Abstract and Conclusion sections of the manuscript.

### 2.3. Targeted Next-Generation Sequencing and Reanalysis of GJB2

Twenty-three study subjects had targeted capture and sequencing of *GJB2* and the previously reported upstream 95 kb critical regulatory region [5]. Agilent SureSelect probes (Agilent Technologies, CA, United States) were used for targeted capture (region size 102.905 kb) with 100% coverage of *GJB2* [(GRCh37/hg19)chr13:20757424-20773956] and 95.99% coverage of the 95 kb critical region [(GRCh37/hg19)chr13:20939400-21034800]. Sequencing was performed using an Illumina MiSeq and a custom bioinformatics pipeline. The NM_004004.5 transcript was used for *GJB2* reanalysis.

### 2.4. ES and Variant Interpretation

Twenty study subjects had ES performed by Novogene Co. (NJ, United States) using the Agilent SureSelect Human All Exon kit (Agilent Technologies, CA, United States) and sequencing on an Illumina platform. Burrows–Wheeler Aligner (BWA) was used to map the paired-end reads to the human reference genome (GRCh37/hg19), SAMtools for sorting the BAM file, Picard to mark duplicate reads, and GATK3.3 variant caller using best practices workflow. Exome data was analyzed using a virtual panel of 119 genes associated with hearing loss (CHOP AUDIOME v 2.3: nonsyndromic HL genes [*n* = 55], syndromic HL genes [*n* = 34], both nonsyndromic and syndromic genes [*n* = 30]) [13–15]. Clinical variant interpretation was performed according to guidelines published by the ClinGen Hearing Loss VCEP [16, 17]. Candidate gene searches performed on exome data included filtering for variants that met the following criteria: (1) *IMPACT = HIGH*; (2) genes with compound heterozygous, homozygous, or hemizygous variants; and (3) the Top 50 ranked variants based on an in-house ranking algorithm that incorporates multiple gene, phenotype, and variant features, with an allele frequency threshold from gnomAD v2.1.1 of < 0.0007 for potential recessive-acting or dominant-acting genes [16]. The candidate gene search included reviewing literature and public databases (PubMed, IMPC, and OMIM) for gene association with hearing-related phenotypes.

After variant curation, cases had four possible outcomes: a diagnosis was made when two pathogenic/likely pathogenic (P/LP) variants were detected in an ARHL gene, a likely diagnostic classification was made when a P/LP and a variant of uncertain significance (VUS) were observed in ARHL gene, an inconclusive case result was determined when two VUS were observed in ARHL gene, and a negative case result had no evidence of two variants in an ARHL gene. Heterozygosity for a P/LP variant in an autosomal dominant hearing loss gene (ADHL) was not observed in this cohort.

### 2.5. GS

PCR-free GS was performed at the BROAD Institute on a subset of 15 individuals with BSNHL without genetic diagnosis by ES. FASTQ files were processed through the DRAGEN Germline Pipeline v3.6 (Illumina, San Diego, CA), which provides CRAM files for viewing variants and multiple VCF files for SNV and CNV outputs. Subsequent sequence and CNVs were filtered and prioritized using an in-house workflow developed in the Division of Genomic Diagnostics at the Children's Hospital of Philadelphia. Sequence variants were scored using a linear weighted sum model with features such as allele frequency in the public genomic database gnomAD (v3), intolerance to loss-of-function variants (LOEUF), computational prediction of potential splicing aberration (SpliceAI), conservation (GERP), and known variants reported in mutation databases such as ClinVar and HGMD. Variants were also assigned a flag for putative recessive variants (Score_R1) based on rarity, conservation, and deleteriousness.

A candidate gene search was also performed to look for novel hearing loss–related genes in cases where GS did not detect a diagnosis (*n* = 12). Candidate gene search examined Exomiser ranked gene lists of potentially dominant acting genes by reviewing the Top 30 hits ranked by *Score_D*, all genes under the recessive filter, *Score*_*R*1 = 1, and the Top 30 genes filtered on HPO term *hearing loss*. Similar to the ES candidate gene search, a population allele frequency cutoff (gnomAD v4.1.0) of < 0.0007 was used for potentially recessive or dominant acting genes. Filtered CNVs were also examined for genes with a known hearing loss association or candidate genes with potential hearing-related phenotypes.

## 3. Results

**Figure 1:**
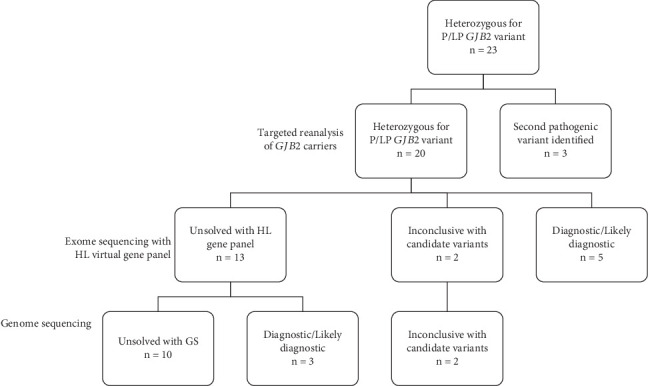
Flow chart of patient diagnoses made with targeted reanalysis of *GJB2*, exome sequencing with HL gene-focused analysis, and genome sequencing.

A retrospective cohort, enrolled from 2003 to 2011, consisting of 23 individuals with BSNHL and heterozygosity for an apparently pathogenic variant in *GJB2*, underwent resequencing and variant reanalysis of *GJB2* (Figure 1 and Table S1). Biallelic P/LP variants were identified in three individuals (Table 1), thus precluding further exome or GS on those study subjects.

The remaining 20 individuals underwent ES with virtual panel analysis of 119 hearing loss–related genes (Figure 1). A diagnostic case result was found in 4/20 individuals. Affected genes in these individuals included *MYO7A* (OMIM #600060), *TMPRSS3* (OMIM #601072), *PDZD7* (OMIM #618003), and *TMC1* (OMIM #600974) (Table 2). All four genes are associated with nonsyndromic, autosomal recessive deafness and have been previously reported in patients with hearing loss [8]. A likely diagnosis was made in one individual with a pathogenic truncating variant and missense VUS in the ARHL gene, *MYO15A* (Table 1). Overall, 5/20 patients who were carriers of a pathogenic *GJB2* variant had an alternative genetic diagnosis associated with their hearing loss.

Two of the 20 cases were classified as inconclusive after virtual panel analysis (Table 2). Study subject HLS-31 showed an apparently homozygous VUS in the *TECTA* gene (c.2657A>G, p.Asn886Ser). There is evidence indicating *TECTA* as causative of ADHL and ARHL (OMIM #601543 and #603629). Although the p.Asn886Ser variant has been reported in individuals with hearing loss [18, 19], it is found at a minor allele frequency in the population that exceeds thresholds for pathogenicity (gnomAD v2.1.1, 0.04%) and can sometimes be found in linkage disequilibrium with a rare intronic deletion (c.5383+5_5383+8del), which is expected to abolish normal splicing and result in loss of function (SpliceAI score of 0.98) [18, 20]. This intronic deletion, though, was not detected in this individual upon additional visual inspection of the NGS data. The inconclusive case, HLS-180, revealed two novel VUSs in the ARHL gene, *CDH23. CDH23* c.6089A>G, p.Glu2030Gly is found at a very low minor allele frequency in the gnomAD v2.1.1 population database (0.0008%) and has a REVEL score of 0.67, which indicates it is likely damaging to protein function. The second VUS detected in this gene, c.8011G>A, p.Gly2671Ser, has a REVEL score of 0.83 and is absent from gnomAD v2.1.1. Neither *CDH23* variant has been previously reported to be associated with disease in the literature at this time.

When including the two inconclusive cases, 15/20 individuals with a heterozygous pathogenic *GJB2* variant remained without a genetic diagnosis after ES with HL gene panel analysis (Figure 1). GS performed on these 15 individuals revealed presumptive diagnoses in three additional individuals (Table 3). Subject HLS-168 had an 8.5 kb intragenic deletion in *LOXHD1* (OMIM #613079), which resulted in loss of Exons 15–17. The deletion was supported by 17 paired-reads with insert size greater than expected and 14 breakpoint spanning split-reads supporting the breakpoints. The breakpoints on both sides fell in SINE/Alu repeats with 8 bp microhomology between them. Both the read-depth caller and the split-read caller identified this event. Variant analysis from GS and ES also revealed a pathogenic heterozygous *LOXHD1* nonsense variant (p.Arg1572∗) in this patient, which is suspected to be *in trans* (Exon 30).

The second finding by GS was a homozygous deletion identified in *STRC* (NM_153700.2) affecting the C-terminal region of the gene and included Exons 19–29 by the read-depth caller and part of the adjacent *CKMT1B* gene (hg38:chr15:43600850-43603898). The split-read caller did not identify the deletion, as there are no paired-end or split reads supporting the event in this highly homologous region. This finding is significant because this gene exists within a segmental duplication and has a nearby pseudogene that prohibits testing by most next-generation sequencing methods. The inclusion of Exons 23–25 within the deleted region is notable since this portion of the gene contains DNA sequence that can be uniquely mapped to *STRC* and not the *STRC* pseudogene [21].

**Figure 2:**
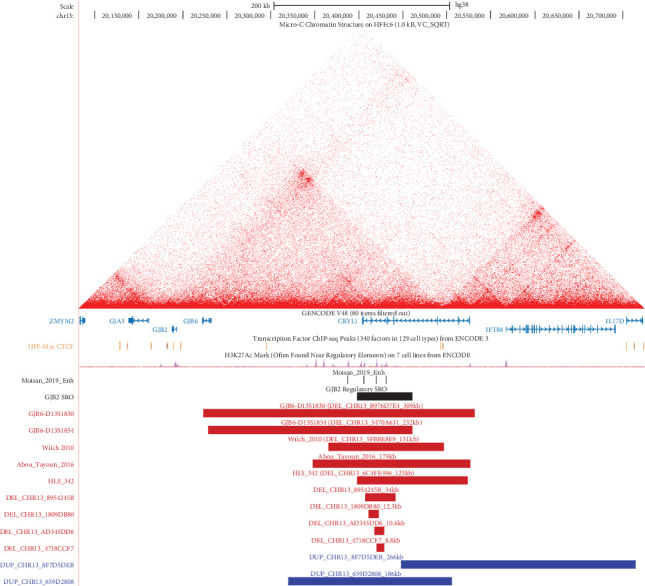
Recurrent deletions and duplications, regulatory elements, and 3D chromatin structure of the DFNB1 locus. Deletions and duplications are named according to the publication in which they were first reported and/or their gnomAD structural variant ID. The chromatin structure of the DFNB1 locus shows it forms a loop mediated by CTCF binding sites that are proximal to *GJB2* and *CRYL1* genes. Image was generated using the UCSC genome browser.

Finally, GS showed the patient that HLS-342 had a ~125 kb heterozygous deletion identified in *CRYL1* (NM_015974.3), impacting Exons 2–8 (Figure 2), and a heterozygous pathogenic variant in *GJB2*, p.Val37Ile. This event was identified by both read-depth and split-read callers with 19 supporting paired-end reads with insert size greater than expected, and seven split-reads spanned the breakpoints (hg38:chr13:20398368-20523822). The deletion sits upstream of the *GJB2* gene and partially overlaps other well-characterized deletions known to affect *GJB2* expression, including GJB6-D13S1830 and GJB6-D13S1854. The 125 kb deletion observed in this patient is found in the gnomAD v4.10 SV database (ID: DEL_CHR13_6C4FE496) with an allele frequency of 0.0084% in non-Finnish European, 0.024% in East Asian, and 0.031% in admixed American populations (Figure 2). When comparing this deletion relative to other reported deletions in this region, we found that it partially maps to the distal end of the 95 kb critical region [5], further refining the smallest region of overlap (SRO) to a 62 kb region impacting the *CRYL1* gene (Figure 2) (coordinates for SRO, hg38:chr13:20398368-20460616). Notably, like other reported upstream *GJB2* deletions, the 125 kb deletion does not impact *GJB6* [5, 6], in line with reports showing no evidence of digenic interaction between *GJB2* and *GJB6*. Smaller deletions overlapping the 125 kb deletion are also found at low allele frequencies in the gnomAD v4.10 SV database (Figure 2 and Table S2) [22]. It is unclear if these smaller deletions are also loss of function variants.

Candidate gene searches were performed on the remaining 12 patients without a diagnosis after GS; however, potential pathogenic variants in genes with strong evidence for hearing loss–related phenotypes in model systems or human biology were not identified. Interestingly, we observed heterozygous P/LP variants in a second (not *GJB2*) ARHL gene in 33% (4/12) of these undiagnosed cases; however, GS analysis did not reveal potentially damaging CNVs affecting these loci directly or indirectly. Thus, it is not uncommon for individuals with hearing loss to be carriers of pathogenic variants in more than one ARHL gene.

## 4. Discussion

This study performed *GJB2* resequencing and variant reanalysis, ES, and GS on a retrospective cohort of 23 individuals with hearing loss and known heterozygosity for an apparently pathogenic variant in *GJB2*. This combined effort yielded 11 new diagnoses (48% diagnostic yield), including detection of a recently characterized, small upstream deletion of the *GJB2* locus. We found that targeted reanalysis of *GJB2* variant carriers led to a second pathogenic variant being identified in three individuals. Given the historical nature of this cohort, it is unclear if the additional pathogenic variants identified in *GJB2* were missed due to technical limitations of prior *GJB2* testing or if these variants were detected but not interpreted as deleterious. Improving diagnostic yield with periodic variant reanalysis has been demonstrated in ES/GS cohorts for rare diseases [23, 24] and also for hearing loss patients [25], mainly due to the discovery of new disease genes or previously seen variants being reclassified as pathogenic. Our work further supports the added value of performing genetic reanalysis, even on a single gene, when patients were tested decades ago.

Analyzing ES data using a virtual panel of hearing loss genes uncovered an additional five diagnoses in ARHL genes, with three individuals impacted by apparently biallelic P/LP variants in *MYO7A*, *TMC1*, and *TMPRSS3*, respectively, and two individuals carrying a VUS and P/LP variant in *PDZD7* and *MYO15A*. Analyzing GS data on the remaining unsolved 15 cases made three additional diagnoses with SNVs and CNVs affecting *LOXHD1*, *STRC*, and *GJB2*. Altogether, this comprehensive genomic analysis found seven different genetic diagnoses of hearing loss in our cohort of *GJB2* pathogenic variant carriers (excluding *GJB2*). These seven genes are among the most prevalent causes of ARHL and are collectively responsible for 31% of total genetic diagnoses for hearing loss [19]. Excluding *GJB2* variant reanalysis, the total diagnostic yield attributable to ES/GS sequencing in our cohort of *GJB2* carriers was 34.7% (8/23). The overall diagnostic rate for hearing loss gene panels ranges from 39% to 48% [14, 19, 26]. Importantly, GS appears to be the most comprehensive testing strategy for hearing loss patients as it detects all variants identified by targeted resequencing and ES as well as additional CNVs, which were missed by these assays.

Identifying comparable diagnostic rates with our cohort of *GJB2* carriers due to pathogenic variants in other ARHL genes highlights the large locus heterogeneity that drives nonsyndromic hearing loss and the substantial *GJB2* carrier frequency in the general population. Indeed, the calculated P/LP carrier frequency in the gnomAD v2.1.1 dataset is approximately 2.7% in all populations using allele frequencies and variants reported as P/LP from ClinVar and Xiang et al. [2]. This estimate remains in line with historical accounts of *GJB2* carrier frequency of ~3% in the US population [27]. Notably, *GJB2* carrier frequency is much higher in East Asian populations due to the p.Val37Ile variant, which alone is present in 8.3% of individuals with this genetic ancestry [28]. Previous reports have called attention to an apparently increased prevalence of *GJB2* carriers relative to the general population in probands that undergo genetic testing for hearing loss and test negative [5, 14]. For instance, in the study by Yamamoto et al., they found *GJB2* carriers comprised 10% of their negative cohort [14]. It remains unclear to what extent these observations are driven by enrichment of genetic ancestries with higher *GJB2* carrier frequencies in their test populations.

Another possibility is that a second variant affecting the *GJB2* locus is missed by conventional gene panel or ES. In this scenario, the most likely culprit would be a deletion impacting the region upstream of *GJB2*. mRNA analysis from patients with one of the four previously reported upstream deletions showed silencing of the *in cis GJB2* allele, most likely due to the elimination of distal enhancer elements that promote *GJB2* expression. The four overlapping deletions include a 179 kb loss reported by Tayoun et al. [5], a 131 kb loss reported by Wilch et al. [6], and 309 kb (GJB6-D13S1830) and 232 kb (GJB6-D13S1854) deletions reported by del Castillo et al. [3, 4]. Altogether, these four deletions have 95 kb of overlap with each other, and functional studies have shown this 95 kb interval encodes at least four enhancer elements that are distal to *GJB2* but in close proximity to neighboring *CRYL1* [29]. Interestingly, chromatin contact mapping studies show that *GJB2* and *CRYL1* are in close physical proximity via chromatin looping, thus further supporting a model whereby distal enhancer elements help activate *GJB2* expression [29]. Using an ethnically diverse cohort, we identified a 125 kb deletion (DEL_CHR13_6C4FE496) that overlaps these previously reported deletions that lie upstream of the *GJB2* locus and define a 62 kb region that intersects the *CRYL1* gene. The 62 kb critical region includes three out of four of the previously identified putative enhancer elements [29]. In addition, the 125 kb deletion eliminates CTCF binding sites that enforce chromatin contact domain formation. While this work was in preparation, a study identified a similar deletion in several patients of East Asian ancestry, further corroborating our findings [30]. Therefore, this deletion is expected to abolish normal chromatin looping and distal enhancer/promoter interactions that occur at this locus. According to the gnomAD population database, this deletion is particularly enriched in East Asian and Latino/admixed American populations; thus, awareness of this variant is likely to improve diagnostic outcomes in these patients. The other previously reported deletions in this region are larger than the 125 kb deletion reported here and are similarly expected to abolish normal distal enhancer/promoter interactions, although the GJB6-D13S1854 does not extend to include the CTCF binding sites present in *CRYL1*. Interestingly, four smaller deletions within the 62 kb region are noted in the general population at low minor allele frequencies (Figure 2 and Table S2). These smaller deletions overlap different combinations of the three enhancer elements that have been previously studied; therefore, their potential identification in patients with hearing loss may further refine this critical regulatory region. Another possible mechanism of inactivation of this regulatory domain could be partially overlapping inverted tandem duplications that generate different chromatin looping structures by placing CTCF binding motifs in different positions and orientations. Such duplications have been reported in other disorders [31] and could abolish the normal enhancer–promoter interactions that are necessary for *GJB2* expression. A 266 and 186 kb duplication are reported in the gnomAD database, which could potentially mediate abnormal positional effects in this region (Figure 2 and Table S2).

Although our data supports the use of short-read GS as a first-line clinical test for BSNHL, approximately half of our cohort (12/23) remained undiagnosed. One approach that could further improve diagnostic yield in hearing loss patients is long-read GS. Advantages of long-read sequencing include identification of copy neutral SVs (inversions, balanced insertions, and translocations), detection of large and small CNVs with accurate breakpoint mapping including within regions of high homology or repetitive elements, methylation profiling, and phasing two variants in autosomal recessive genes to uncover distinct haplotypes [32]. Long-read GS has been shown in several studies of undiagnosed patients with suspected rare diseases to result in additional diagnoses [32–38]. Furthermore, the implementation of long-read GS as a first-line clinical test has provided expanded variant detection and faster turnaround relative to standard of care testing that typically follows a sequential or combinatorial testing strategy of short-read sequencing, microarray, and other targeted variant assays [38]. Given the complexity of testing for hearing loss–related genes, studies evaluating long-read sequencing in this patient population will be of future interest.

One of the limitations of our study is the lack of CNV analysis in our ES data. This is in part due to limitations in the design of this study and the lack of run controls and intrarun comparator samples to accurately call deletions and duplications by exon read depth [39]. Detecting copy number variants in *STRC* is challenging due to the presence of the *STRCP* pseudogene; thus, we suspect that the deletion observed in our patient may not have been easily detected by ES; however, we believe that most laboratories performing ES copy number analysis would have detected the *LOXHD1* deletion. The 125 kb deletion impacting *CRYL1* would most likely be overlooked by ES since this is not a known hearing loss gene and ES cannot provide the positions of breakpoints, thus prohibiting the identification of this CNV in databases such as gnomAD. Other limitations of our study include the restricted clinical data available for this research cohort, which is partly due to the historical nature of this sample set. Additionally, parental testing was not performed to validate biallelic segregation of variants in ARHL genes. However, we note that the majority of the variants reported here have been previously described in the literature without evidence of segregating in cis with other variants.

## 5. Conclusion

When combining the data generated from the sequential testing strategy performed on this cohort, our results strongly support that GS is a first-tier test for patients with BSNHL and as a follow-up test in patients that remain undiagnosed after a history of less comprehensive testing. All findings by *GJB2* targeted resequencing and ES would have been captured by GS, which in turn also led to the identification of clinically important deletions that were not revealed by the former assays. Since GS inherently provides a more complete evaluation of genetic variation relative to ES, including the enhanced ability to detect novel single nucleotide, copy number, and SVs in coding and noncoding regions, its main limitation with regard to future adoption by clinical laboratories will most likely be a lack of affordability and reimbursement. However, the recent falling costs associated with short-read sequencing are increasingly making it clear that GS can be adopted as a first-tier test for BSNHL as has been recommended for other disorders such as pediatric patients with congenital anomalies or intellectual disability [40].

## Figures and Tables

**Table 1:** Cases solved by *GJB2* reanalysis and sequencing.

**Case ID**	**Genomic pos. (GRCh38)**	**Type**	**Zygosity**	**cDNA**	**Protein**	**Class**	**ACMG/AMP criteria**	**PMID**
HLS-630	chr13:g.20189548del	Frameshift	Het	c.35delG	p.Gly12Valfs	P	PVS1, PM3_VS, PS4, and BA1	16380907
	chr13:g.20189481A>C	Missense	Het	c.101T>G	p.Met34Arg	P^a^	PS4, PM3_VS, PP1_S, PM5, and PS3_M	31160754 and 30311386
HLS-582	chr13:g.20189548del	Frameshift	Het	c.35delG	p.Gly12Valfs	P	PVS1, PM3_VS, PS4, and BA1	16380907
	chr13:g.20189605T>G	Splice	Het	c.-22-2A>C		LP^a^	PM3_VS, PP1, PS3_P, and BS1	25401782, 24039984, and 33096615
HLS-055	chr13:g.20189548del	Frameshift	Het	c.35delG	p.Gly12Valfs	P	PVS1, PM3_VS, PS4, and BA1	16380907
	chr13:g.20192782C>T	Splice	Het	c.-23+1G>A		P^a^	PVS1, PS3_P, PM3_VS, and PM2	11935342, 27224056, and 26096904

Abbreviations: Het, heterozygous; LP, likely pathogenic; P, pathogenic.

^a^Detected after reanalysis.

**Table 2:** Exome sequencing results from heterozygous carriers of pathogenic *GJB2* variants.

**Case ID**	**Gene**	**Genomic pos. (GRCh38)**	**Type**	**Zygosity**	**cDNA**	**Protein**	**Class**	**ACMG/AMP criteria**	**PMID**
Likely diagnostic cases									
HLS-88	MYO7A NM_000260.3	chr11:g.77160271G>A	Missense	Het	c.1189G>A	p.Ala397Thr	LP	PM2, PM3_S, PP3, and PP4	23591405 and 28944237
		chr11:g.77203120del	Frameshift	Het	c.5229delG	p.Leu1744Cysfs∗61	LP	PM2 and PVS1	
HLS-625	TMC1 NM_138691.2	chr9:g.72754817C>T	Missense	Het	c.674C>T	p.Pro225Leu	LP	PM2, PM3_S, and PP4	33111345 and 34523024
		chr9:g.72816213A>G	Splice	Het	c.1763+3A>G		P	PP3, PM3_S, PP1, and PS3	21252500
HLS-37	TMPRSS3 NM_024022.2	chr21:g.42375787C>T	Missense	Het	c.1276G>A	p.Ala426Thr	P	PM3_S, PP1_S, PP3, and PS3_P	21786053, 28566687, and 29196752
		chr21:g.42380136C>G	Missense	Het	c.1029G>C	p.Trp343Cys	LP	PM2, PP3, PM3_P, and PM5_S	32279305, 32860223, and 25770132
HLS-316	PDZD7 NM_001195263.1	chr10:g.101009275_ 101009276insC	Frameshift	Het	c.2692dupG	p.Ala898Glyfs∗63	LP	PM2 and PVS1	
		chr10:g.101021859A>C	Missense	Het	c.806T>G	p.Ile269Ser	VUS	PM2 and PM3_S	37086329
HLS-138	MYO15A NM_016239.3	chr17:g.18138226G>A	Missense	Het	c.4987G>A	p.Asp1663Asn	VUS	PM2 and PP3	
		chr17:g.18156953G>A	Splice	Het	c.8602-1G>A		P	PVS1, PM2, and PP3	
Inconclusive cases									
HLS-180	CDH23 NM_022124.5	chr10:g.71791171A>G	Missense	Het	c.6089A>G	p.Glu2030Gly	VUS	PM2 and PP3	
		chr10:g.71805944G>A	Missense	Het	c.8011G>A	p.Gly2671Ser	VUS	PM2 and PP3	
HLS-31	TECTA NM_005422.2	chr11:g.121129927A>G	Missense	Hmz	c.2657A>G	p.Asn886Ser	VUS	PM3_S, BS1_P, and BP2	21520338, 27068579, and 29196752

Abbreviations: Het, heterozygous; Hmz, homozygous; LP, likely pathogenic; P, pathogenic; VUS, variant of uncertain significance.

**Table 3:** Likely diagnostic genome sequencing results from heterozygous carriers of pathogenic *GJB2* variants.

**Case ID**	**Gene**	**Genomic pos. (GRCh38)**	**Type**	**Zygosity**	**cDNA**	**Protein**	**Class**	**ACMG/AMP criteria**	**PMID**
HLS-168	LOXHD1 NM_144612.6	chr18:g.46524734G>A	Nonsense	Het	c.4714C>T	p.Arg1572∗	P	PVS1, PM3_S, and PP1_S	19732867, 21465660, and 25792669
		chr18:g.46564926_46573436del	Deletion	Het			P	PVS1 and PM2	
HLS-240	STRC NM_153700.2	chr15:g.43600850_43603898del	Deletion	Hmz			P		25157971
HLS-342	GJB2 NM_004004.5	chr13:g.20189473C>T	Missense	Het	c.109G>A	p.Val37Ile	P	PS4, PP1_S, and PM3	31160754 and 30311386
	CRYL1 NM_015974.3	chr13:g.20398368_20523822del	Deletion	Het			VUS		

Abbreviations: Het, heterozygous; Hmz, homozygous; P, pathogenic; VUS, variant of uncertain significance.

## Data Availability

The data that support the findings of this study are available from the corresponding authors upon reasonable request.

## Supporting Information

Supporting Information Additional supporting information can be found online in the Supporting Information section. Table S1: Patient cohort and clinical characteristics. Table S2: Copy number variants intersecting *GJB2* regulatory SRO.

## References

[1] Mitchell C. O., Morton C. C. (2021). Genetics of Childhood Hearing Loss. *Otolaryngologic Clinics of North America*.

[2] Xiang J., Sun X., Song N., Ramaswamy S., Abou Tayoun A. N., Peng Z. (2023). Comprehensive Interpretation of Single-Nucleotide Substitutions in GJB2 Reveals the Genetic and Phenotypic Landscape of GJB2-Related Hearing Loss. *Human Genetics*.

[3] del Castillo I., Villamar M., Moreno-Pelayo M. A. (2002). A Deletion Involving the Connexin 30 Gene in Nonsyndromic Hearing Impairment. *New England Journal of Medicine*.

[4] del Castillo F. J., Rodríguez-Ballesteros M., Alvarez A. (2005). A Novel Deletion Involving the Connexin-30 Gene, del(GJB6-d13s1854), Found in Trans With Mutations in the GJB2 Gene (Connexin-26) in Subjects With DFNB1 Non-Syndromic Hearing Impairment. *Journal of Medical Genetics*.

[5] Tayoun A. N., Mason-Suares H., Frisella A. L. (2016). Targeted Droplet-Digital PCR as a Tool for Novel Deletion Discovery at the DFNB1 Locus. *Human Mutation*.

[6] Wilch E., Azaiez H., Fisher R. A. (2010). A Novel DFNB1 Deletion Allele Supports the Existence of a Distant Cis-Regulatory Region That Controls GJB2 and GJB6 Expression. *Clinical Genetics*.

[7] DiStefano M. T., Hemphill S. E., Oza A. M. (2019). ClinGen Expert Clinical Validity Curation of 164 Hearing Loss Gene-Disease Pairs. *Genetics in Medicine*.

[8] Azaiez H., Booth K. T., Ephraim S. S. (2018). Genomic Landscape and Mutational Signatures of Deafness-Associated Genes. *American Journal of Human Genetics*.

[9] Abbasi W., French C. E., Rockowitz S., Kenna M. A., Eliot Shearer A. (2022). Evaluation of Copy Number Variants for Genetic Hearing Loss: A Review of Current Approaches and Recent Findings. *Human Genetics*.

[10] Shearer A. E., Kolbe D. L., Azaiez H. (2014). Copy Number Variants Are a Common Cause of Non-Syndromic Hearing Loss. *Genome Medicine*.

[11] Rentas S., Abou Tayoun A. (2021). Utility of Droplet Digital PCR and NGS-Based CNV Clinical Assays in Hearing Loss Diagnostics: Current Status and Future Prospects. *Expert Review of Molecular Diagnostics*.

[12] Kim B. J., Oh D. Y., Han J. H. (2020). Significant Mendelian Genetic Contribution to Pediatric Mild-to-Moderate Hearing Loss and Its Comprehensive Diagnostic Approach. *Genetics in Medicine*.

[13] Guan Q., Balciuniene J., Cao K. (2018). AUDIOME: A Tiered Exome Sequencing-Based Comprehensive Gene Panel for the Diagnosis of Heterogeneous Nonsyndromic Sensorineural Hearing Loss. *Genetics in Medicine*.

[14] Yamamoto N., Balciuniene J., Hartman T. (2023). Comprehensive Gene Panel Testing for Hearing Loss in Children: Understanding Factors Influencing Diagnostic Yield. *Journal of Pediatrics*.

[15] Sheppard S., Biswas S., Li M. H. (2018). Utility and Limitations of Exome Sequencing as a Genetic Diagnostic Tool for Children With Hearing Loss. *Genetics in Medicine*.

[16] Oza A. M., DiStefano M. T., Hemphill S. E. (2018). Expert Specification of the ACMG/AMP Variant Interpretation Guidelines for Genetic Hearing Loss. *Human Mutation*.

[17] Abou Tayoun A. N., Pesaran T., DiStefano M. T. (2018). Recommendations for Interpreting the Loss of Function PVS1 ACMG/AMP Variant Criterion. *Human Mutation*.

[18] Hildebrand M. S., Morín M., Meyer N. C. (2011). DFNA8/12 Caused by TECTA Mutations Is the Most Identified Subtype of Nonsyndromic Autosomal Dominant Hearing Loss. *Human Mutation*.

[19] Sloan-Heggen C. M., Bierer A. O., Shearer A. E. (2016). Comprehensive Genetic Testing in the Clinical Evaluation of 1119 Patients With Hearing Loss. *Human Genetics*.

[20] Lezirovitz K., Batissoco A. C., Lima F. T. (2012). Aberrant Transcript Produced by a Splice Donor Site Deletion in the TECTA Gene Is Associated With Autosomal Dominant Deafness in a Brazilian Family. *Gene*.

[21] Amr S. S., Murphy E., Duffy E. (2018). Allele-Specific Droplet Digital PCR Combined With a Next-Generation Sequencing-Based Algorithm for Diagnostic Copy Number Analysis in Genes With High Homology: Proof of Concept Using Stereocilin. *Clinical Chemistry*.

[22] Collins R. L., Brand H., Karczewski K. J. (2020). A Structural Variation Reference for Medical and Population Genetics. *Nature*.

[23] Wenger A. M., Guturu H., Bernstein J. A., Bejerano G. (2017). Systematic Reanalysis of Clinical Exome Data Yields Additional Diagnoses: Implications for Providers. *Genetics in Medicine*.

[24] Liu P., Meng L., Normand E. A. (2019). Reanalysis of Clinical Exome Sequencing Data. *New England Journal of Medicine*.

[25] Sun Y., Xiang J., Liu Y. (2019). Increased Diagnostic Yield by Reanalysis of Data From a Hearing Loss Gene Panel. *BMC Medical Genomics*.

[26] Baux D., Vaché C., Blanchet C. (2017). Combined Genetic Approaches Yield a 48% Diagnostic Rate in a Large Cohort of French Hearing-Impaired Patients. *Scientific Reports*.

[27] Green G. E., Scott D. A., McDonald J. M., Woodworth G. G., Sheffield V. C., Smith R. J. (1999). Carrier Rates in the Midwestern United States for GJB2 Mutations Causing Inherited Deafness. *JAMA*.

[28] Shen J., Oza A. M., Del Castillo I. (2019). Consensus Interpretation of the p.Met34Thr and p.Val37Ile Variants in GJB2 by the ClinGen Hearing Loss Expert Panel. *Genetics in Medicine*.

[29] Moisan S., Le Nabec A., Quillévéré A., Le Maréchal C., Férec C. (2019). Characterization of GJB2 Cis-Regulatory Elements in the DFNB1 Locus. *Human Genetics*.

[30] Lin Z., Xiang J., Sun X. (2024). Genome Sequencing Unveils the Role of Copy Number Variants in Hearing Loss and Identifies Novel Deletions With Founder Effect in the DFNB1 Locus. *Human Mutation*.

[31] D'haene E., Vergult S. (2021). Interpreting the Impact of Noncoding Structural Variation in Neurodevelopmental Disorders. *Genetics in Medicine*.

[32] Eisfeldt J., Ek M., Nordenskjöld M., Lindstrand A. (2025). Toward Clinical Long-Read Genome Sequencing for Rare Diseases. *Nature Genetics*.

[33] Sinha S., Rabea F., Ramaswamy S. (2025). Long Read Sequencing Enhances Pathogenic and Novel Variation Discovery in Patients With Rare Diseases. *Nature Communications*.

[34] Showpnil I. A., E Hernandez Gonzalez M., Ramadesikan S. (2024). Long-Read Genome Sequencing Resolves Complex Genomic Rearrangements in Rare Genetic Syndromes. *NPJ Genomic Medicine*.

[35] Mizuguchi T., Suzuki T., Abe C. (2019). A 12-kb Structural Variation in Progressive Myoclonic Epilepsy Was Newly Identified by Long-Read Whole-Genome Sequencing. *Journal of Human Genetics*.

[36] Sone J., Mitsuhashi S., Fujita A. (2019). Long-Read Sequencing Identifies GGC Repeat Expansions in NOTCH2NLC Associated With Neuronal Intranuclear Inclusion Disease. *Nature Genetics*.

[37] Miller D. E., Sulovari A., Wang T. (2021). Targeted Long-Read Sequencing Identifies Missing Disease-Causing Variation. *American Journal of Human Genetics*.

[38] Thiffault I., Farrow E., Barrett C. (2025). Clinical Long-Read Sequencing Test for Genetic Disease Diagnosis. *JAMA Pediatrics*.

[39] Rajagopalan R., Murrell J. R., Luo M., Conlin L. K. (2020). A Highly Sensitive and Specific Workflow for Detecting Rare Copy-Number Variants From Exome Sequencing Data. *Genome Medicine*.

[40] Manickam K., McClain M. R., Demmer L. A. (2021). Exome and Genome Sequencing for Pediatric Patients With Congenital Anomalies or Intellectual Disability: An Evidence-Based Clinical Guideline of the American College of Medical Genetics and Genomics (ACMG). *Genetics in Medicine*.

